# Factors Associated with Adherence to Clinical Practice Guidelines for Patients with Type 2 Diabetes Mellitus: Results of a Spanish Delphi Consensus

**DOI:** 10.1155/2021/9970859

**Published:** 2021-10-23

**Authors:** José Antonio Gimeno, Gloria Cánovas, Alejandra Durán

**Affiliations:** ^1^Service of Endocrinology, Hospital Clínico Lozano Blesa, Zaragoza, Spain; ^2^Service of Endocrinology, Hospital de Fuenlabrada, Madrid, Spain; ^3^Service of Endocrinology, Hospital Clínico San Carlos, Madrid, Spain

## Abstract

**Objective:**

To assess factors associated with adherence to clinical practice guidelines (CPGs) for type 2 diabetes mellitus (T2DM).

**Methods:**

A cross-sectional multicenter study based on a two-round Delphi survey was designed. A total of 98 endocrinologists (mean age 45 years) involved in the care of T2DM patients completed a 43-item questionnaire assessing different aspects of adherence related to CPGs.

**Results:**

Most participants worked in tertiary care public hospitals. All participants used CPGs, with ADA/EASD as the most common (99%). The lack of time, establishment of an individualized management of patients, insufficient human resources, and therapeutic inertia were scored as the main reasons for not following CPGs recommendations. Participants agreed that insufficient material resources and limitations established by the healthcare system prevent adherence to CPGs. The risk of hypoglycemia was considered to be limiting factor for the patients' integral control. Also, there was consensus on the need to have the support of nursing personnel with specific training in diabetes as well as dietitians and podiatrists. There was disagreement regarding the influence on adherence to CPGs of patient's characteristics not matching those of CPGs, patient's preferences, tolerability of the action recommended, concomitant comorbidities, or pluripathological conditions. Differences according to the participant's age (≤40 years vs. >40 years) were not found. Therapeutic inertia and lack of time did not show a significant correlation.

**Conclusions:**

Nonadherence to CPGs on T2DM is a multifactorial problem but the existence multiple CPGs, the lack of time, the therapeutic inertia, and insufficient human resources have been identified as factors limiting adherence. Hypoglycemia continues to be a barrier for achievement of targets recommended by CPGs.

## 1. Introduction

Diabetes mellitus (DM) is a complex multisystemic disease that requires high quality care, increases the risk of potentially life-threatening complications, and imposes a high impact on healthcare services and a heavy economic burden on society [1–3]. According to estimates of the global prevalence of diabetes for 2000, projections extrapolated for 2030 to all 191 World Health Organization (WHO) member states, the prevalence of the disease is projected to rise from 2.8% to 4.4%, the total number of people with DM from 171 million to 366 million, with an increase in the proportion of diabetic people >65 years, and the urban population with DM in developing countries to double [4]. Therefore, in the presence of these negative perspectives, strategies for improving effective diagnosis and management of subjects with DM are of utmost importance.

The pathogenesis and long-term metabolic and vascular complications of type 2 diabetes (T2DM) are fairly well known but its treatment has remained challenging, with only half of the patients achieving the recommended HbA1c target, and a significant proportion of the diabetic population still do not receiving adequate care [5]. Clinical practice guidelines (CPGs) provide comprehensive evidence-based recommendations based on randomized controlled trials (RCTs) for all relevant aspects of DM, including diagnosis and treatment of type 1 and type 2 in children and adults, strategies for the prevention or delay of T2DM, and therapeutic approaches that can reduce morbidity, in particular, the risk of renal and cardiovascular complications, and improve patient outcomes [6–11]. It has been clearly shown that the use of guidelines is associated with better prognosis, achieving target goals for HbA1c, blood pressure, renal function, and lipid profiles, as well as greater patient's satisfaction than when they are not used [12–14]. However, the adherence of healthcare professionals to recommendations of CPGs is unsatisfactory, with deficiencies in most areas of diabetes care and follow-up [15–19]. Barriers reported include lack of awareness, familiarity or agreement with the content, poor understanding of the need for change to overcome the inertia of normal practice, differences in the goals between clinicians and patients, and a number of external constraints associated with deficiencies in equipment and space, shortage of time, staff, and financial resources [20–23]. It has been emphasized that it is essential to recognize the factors that act as barriers and contribute to the missing link between theory and practice of diabetic guidelines [24], which will help to design and implement appropriate strategies for effective and improved diabetes guidelines adherence and management.

To address these issues, this study was designed with the primary objective of assessing factors involved in adherence to CPGs on T2DM diabetes. Secondary objectives were to identify major barriers preventing adherence to main CPGs and to develop a document with conclusions regarding actions for improving adherence to guidelines, which will finally contribute to a more effective care of patients with T2DM.

## 2. Methods

### 2.1. Study Design

This was a cross-sectional multicenter study based on a two-round Delphi survey (the IMPLICA study). IMPLICA is the Spanish acronym for “Factores IMPLicados en el seguimiento de las guías de páctIca Clínica en diAbetes” (factors involved in adherence to clinical practice guidelines in diabetes). The study was conducted over two 3-month periods (first Delphi round from April to June, 2020; second Delphi round from August to October, 2020). The study protocol was approved by the Ethics Committee for Clinical Research of Hospital Clínico San Carlos, Madrid (Spain).

### 2.2. Participants and Procedures

A panel of three clinically active specialists in endocrinology, metabolism, and nutrition with special interest and large experience in the care of patients with diabetes was recruited to form the scientific committee of the study. They were responsible for the development of the study questionnaire and supervision of the progression of the study, including the recruitment of participants, coordination of the analysis, and interpretation of data. The content and topics to be included in the study questionnaire were designed by the scientific committee based on their experience and a comprehensive search of the literature to identify previously conducted studies with high level of evidence and key primary studies focused on the care of subjects with 2TDM and, particularly, on CPGs and adherence to recommendations.

Study participants were experienced practicing specialists in endocrinology, metabolism, and nutrition attending a minimum of 10 patients with T2DM every week in either public or private institutions throughout Spain. Participants were recruited through formal e-mail invitations that included a brochure with full information about the project. Participation in the study was anonymous. The questionnaire was lodged in an Internet microsite that could be accessed via a weblink included in the brochure. Only physicians who accepted to participate in the study were provided with access to the questionnaire microsite URL and the user's password. Participation in the study was anonymous and voluntary.

The final questionnaire emerged from a two-round Delphi consensus process and was composed of 8 sections with a total of 43 items.  Section 1 (5 items) included data of the investigator and use of CPGs on diabetes, Section 2 (7 items) data on the participant's healthcare center and volume of diabetic patients attended, Section 3 (13 items) factors related to CPGs, Section 4 (3 items) factors related to the healthcare system, Section 5 (3 items) factors related to the healthcare center, Section 6 (2 items) factors related to diabetes, Section 7 (6 items) factors related to the clinician, and Section 8 (7 items) factors related to the patient. There were open questions, multiple choice questions, and some other questions formulated so that they could be answered using a 5-point Likert scale from 1 = ^“^*strongly* *disagree*,^”^2 = ^“^*moderately* *disagree*,^”^3 = ^“^*neither* *agree* *nor* *disagree*^”^ (indifferent), 4 = ^“^*moderately* *agree*,^”^ and 5 = ^“^*strongly* *agree*,^”^ according to the participant's opinion. The study questionnaire is described in the Supplementary material (available here).

### 2.3. Statistical Analysis

For each item of the study questionnaire, the mean value of the five possible responses in the 5-point Likert scale was calculated. A consensus was established in favor of the recommendation when the sum of responses “strongly agree” (Likert score 5), and “moderately agree” (Likert score 4) was greater than 75% of the total responses obtained for that item. By contrast, a consensus against the recommendation was reached when the sum of responses “strongly disagree” (Likert score 2) and “moderately disagree” (Likert score 1) was greater than 75% of the total responses for that item. When none of these previous assumptions were met, a consensus for or against the statement was not reached. Since this was a qualitative rather than a quantitative study, the number of selected participants based on the probabilistic error was not established [25, 26]. Categorical variables are expressed as frequencies and percentages and continuous data as mean and standard deviation (SD) or median and interquartile range (25th-75th percentile). Participants were divided according to age in the groups of ≤40 years and>40 year, and differences in the distribution of variables were analyzed with the Mann–Whitney *U* test for independent samples. The relationship between the lack of time in consultations and the therapeutic inertia was assessed with Spearman's rank-order correlation coefficient (*ρ*). Data were analyzed using the SAS statistical program (Statistical Analysis Systems, SAS Institute, Cary, NC, USA) version 9.1.3 for Windows.

## 3. Results

### 3.1. Participants and Use of CPGs

A total of 98 endocrinologists (40 men, 58 women) with a mean (SD) age of 44.9 (9.2) years (range 29-65) agreed to participate in the study. They had been practicing for a mean (SD) of 16.0 (9.5) years. Almost all participants (90.8%) worked in public hospitals (tertiary care hospitals in 75%) located in urban areas (99%), with a median of 9 endocrinologists working in their services. Participation in a training program on diabetes and in a research program in the previous 12 months was reported by 78.6% and 53.1% of participants, respectively. Also, 67.3% reported that they had attended between 76 and 150 patients in the last week, and that more than 25% of patients were diagnosed with T2DM according to 69.4% of participants.

**Figure 1:**
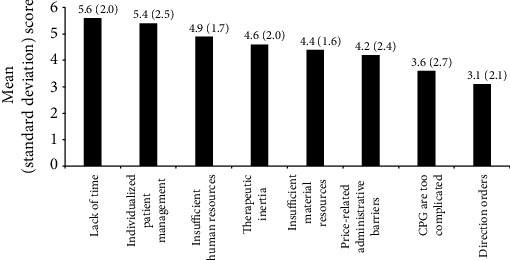
Factors related to not following recommendations of CPGs (scored from 1 = *no* *influence* to 8 = *maximum* *influence*; data as mean and standard deviation in parenthesis).

All participants used CPGs in the care of patients with T2DM, with the American Diabetes Association/European Association for the Study of Diabetes (ADA/EASD) recommendations as the most commonly used guideline (99%), followed by the document of integral approach of T2DM of the Spanish Society of Endocrinology and Nutrition (SEEN) by 79.6%, the American Association of Clinical Endocrinologists/American College of Endocrinologists (AACE/ACE) comprehensive type 2 diabetes management algorithm by 38.8%, the recommendations for the pharmacological treatment of hyperglycemia in T2DM of the Spanish Society of Diabetes (SED) by 34.7%, the T2DM in adults: management NICE by 10.2%, and the Clinical Practice Guidelines for the Prevention and Management of Diabetes in, Canada, Professional Section of Diabetes Canada by 8.2%. As shown in Figure 1, the lack of time, establishment of an individualized management of patients, insufficient human resources, and therapeutic inertia were scored as the main reasons for not following the recommendations of CPGs.

### 3.2. Factors Related to the CPG

Consensus was achieved in 7 of 10 items included in this section (percentages of agreement from 80.2% to 98%) (Table 1). The remaining three items in which consensus was not obtained were “the evolution of the research, given that sometimes there are subsequent findings that are proven uncertain or irreproducible, may reduce the credibility of the CPG recommendations,” “the complexity of the process recommended in the CPG difficults adherence,” and “although a guide is well implemented it is difficult to maintain it, since after a certain time professionals tend to return to their previous routines” with percentages of agreement of 74%, 43,8%, and 25%, respectively. Lack of time, the need to establish individualized treatment, insufficient human resources, therapeutic inertia, and insufficient material resources were the most common factors associated with nonadherence to CPGs (Figure 1).

### 3.3. Factors Related to the Healthcare System and Healthcare Center

Participants agreed that limitations established by the healthcare system prevent adherence to CPGs (75%), and that differences in local administrative regulations between autonomous communities have a different impact on adherence (90.9%) (Table 2). The statement of the lack of coincidence between recommendations of international CPGs and the current situation of the healthcare systems did not achieved consensus (42.7%). In relation to the influence of conditions of the healthcare center, availability of time (92.7%) and human resources (86.5%) were considered to affect adherence, whereas participants did not agree on the influence on the shortage of adequate material resources for diagnosis and treatment of T2DM (54.2%) (Table 2).

### 3.4. Factors Related to Diabetes and the Clinician

As shown in Table 3, participants disagreed that complexity of diabetes affected adherence to CPGs (63.5%) but agreed that the risk of hypoglycemia continues to be a limiting factor for the patients' integral control (83.4%). In relation to the factors associated with the clinician, consensus was reached in 3 of the 6 items (50%), which included the following: “therapeutic inertia means that despite knowing the CPG recommendations, the clinician continues with his previous practice” (83.3%), “the need to have the support of dietitians and podiatrists” (95.9%), and “the insufficient number of nursing personnel with specific training in diabetes education makes it difficult to approach patients with T2DM” (95.9%). Neither regular updating of CPG, nor complexity of the pathology or pharmacological treatment nor problems derived from the lack of connection between all members of the interdisciplinary team, achieved consensus (Table 3).

### 3.5. Factors Related to the Patient

**Figure 2:**
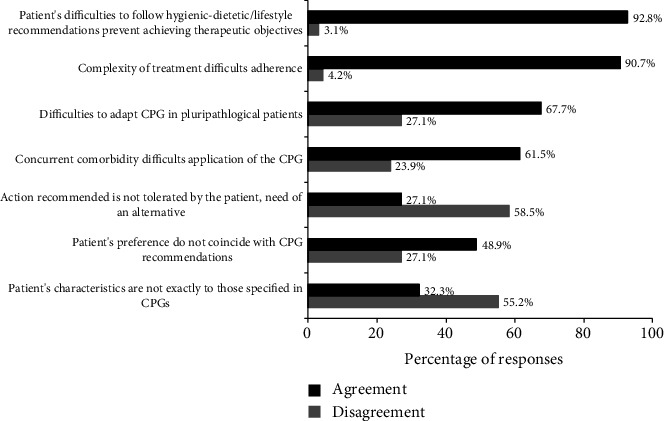
Factors related to the patient preventing adherence to recommendations of CPGs (5-point Likert scale; agree: moderately/strongly agree (scores 4-5), disagree: moderately/strongly disagree (scores 1-2).

In this section of 7 items, consensus was achieved in only 2 (28.6%) (Figure 2), including that complexity of treatment difficults therapeutic adherence (90.7%) and patient's difficulties to follow hygienic-dietetic and lifestyle recommendations prevented to achieve therapeutic objectives (92.8%). In relation to other factors including patient's characteristics not matching those of CPGs, patient's preferences, tolerability of the action recommended, concomitant comorbidities, or pluripathological conditions, participants disagreed on their influence on adherence to CPGs (Figure 2).

Finally, there were no statistically significant differences in the responses obtained according to the participant's age of ≤40 years (*n* = 37) and>40 years (*n* = 61) in eight items selected by the scientific committee, which included “the complexity of the process recommended in the CPG difficults adherence” and “the large number of CPGs on diabetes may prevent effective dissemination” as two factors related to the CPG (Table 1); “insufficient material resources for diagnosis and treatment recommended by CPGs” as one factor related to the healthcare center (Table 2); “complexity of diabetes difficults adherence to CPGs” as one factor related to diabetes (Table 3); “therapeutic inertia favors continuation with previous practice,” “constant updates of the CPGs make it difficult to be up to date and have a deep knowledge of them,” and “professionals must handle complex pharmacological treatment, which is perceived as a difficulty for intensification” as three factors related to the clinician (Table 3); and “complexity of treatment difficults therapeutic adherence” as one factor related to the patient (Figure 2). Moreover, therapeutic inertia was not significantly correlated with the lack of time in consultation (*ρ* = 0.002; *P* = 0.983).

## 4. Discussion

This two-round Delphi study was conducted to explore the level of adherence to CPGs for T2DM by Spanish endocrinologists and to identify barriers associated with nonadherence. Different characteristics of participants enhance the value of their responses, including a wide range of age (mean 45 years), large experience (mean 16 years), tertiary care hospitals from the public health care system and large specialized services (median 9 endocrinologist) as the working setting, and high percentage of endocrinologists who reported participation in training programs of diabetes and research projects during the last year. These characteristics may explain the fact that 100% of participants were aware and used CPGs in daily practice, with ADA/EASD recommendations as the most commonly used by almost all of them, followed by recommendations of the SEEN, the AACE/ACE, and the SED. Other CPGs (NICE and Diabetes Canada) were used by only around 10% of participants. Interestingly, lack of time, individualized patient management, and insufficient human resources were three important reasons to account for nonadherence to CPGs on the endocrinologist perspective.

Although many studies have addressed compliance with metabolic and biochemical targets, blood pressure, healthy diet, physical exercise, or therapeutic recommendations in retrospective and prospective cohorts of subjects with T2DM [27–30], there is little evidence of the physicians' opinions regarding adherence and difficulties for implementing recommendations of CPGs in clinical practice. Several factors have been noted to affect the implementation and adherence to diabetic guidelines, which can be categorized in different major groups, such as factors associated with intrinsic attributes of CPGs themselves, factors related to the implementation process, physician's and patient's related characteristics, and factors related to the organization of the healthcare system [24].

From the perspective of intrinsic features of guidelines, there was a consistent agreement on the advantages of their use, particularly, to support appropriate decisions in individual patients, the need to update information regularly, and the importance of incorporating adherence indicators in the CPGs. In relation to factors associated with clinicians, consensus was achieved regarding insufficient human resources with support on the part of nursing personnel, dietitians, and podiatrists, as well as the risk of hypoglycemia as a limiting factor for the control of patients and the negative role of therapeutic inertia. Therapeutic inertia impairs the ability of to attain and maintain glycemic targets, which in turn increases risks for the development and progression of diabetes-related complications. Therapeutic inertia has been identified as an important contributor to failure to advance or deintensify treatment, although in a broader concept clinical inertia includes also issues such as failure to screen, make appropriate referrals, and manage risk factors and complications [31]. In a systematic review of 53 studies of therapeutic inertia in the treatment of hyperglycemia in patients with T2DM, the median time to treatment intensification after a HbA1c measurement above target was more than 1 year. Therapeutic inertia increased as the number of antidiabetic drugs rose and decreased with increasing HbA1c levels [32]. In a retrospective analysis using electronic medical records from 23,678 patients with HbA1c ≥ 7% and a first prescription for a new noninsulin antidiabetic drug or insulin recorded from January 2010 to December 2014, in Catalonia, Spain, intensification was not undertaken in 1 in 5 patients, with a median time to first intensification of 17.1 months [33].

In a study to assess adherence to German treatment guidelines for T2DM with the participation of 46 experienced physicians, they had a consistent perspective on the value of the national treatment guidelines, but perceived patient inability and demotivation to be the strongest adherence barriers [22]. In the present study, complexity of treatment and patient's difficulties to follow hygienic-dietetic/lifestyle recommendations was remarkable barriers preventing adherence to CPGs. Concurrent comorbidity and pluripathology were also limiting factors. Another interesting finding of the study was the absence of significant differences according to age of the participants, which may indicate that neither age nor the years of experience might have an impact on the causes of nonadherence to CPGs.

The present results should be interpreted taking into account the observational and exploratory nature of the survey based on self-reporting, reflecting the subjective perception of the participants. However, the number of participants was almost 100, and all of them were endocrinologists with a solid experience in type 2 diabetic patients care; so, the survey was able to capture a broad set of perspectives of adherence barriers related to the guidelines, healthcare system, physicians, and patients. It is, to our knowledge, the first assessment of physician perspective on T2DM guideline adherence in the Spanish-speaking area.

## 5. Conclusions

Nonadherence to CPGs on T2DM is a multifactorial problem but the existence of multiple CPGs, the lack of time, the therapeutic inertia, and the complexity of diabetes has been identified as factors limiting adherence. Hypoglycemia continues to be a barrier for achievement of targets recommended by CPGs. Increasing the number of healthcare professionals in the multidisciplinary teams (diabetes specialist nurses, dietitians, and podiatrists) is necessary in providing good patient care and promoting adherence to CPGs.

## Figures and Tables

**Table 1:** Factors related to clinical practice guidelines (CPGs).

Items of the questionnaire	Total number	Number of responses (%)		
		Disagree (moderately disagree/strongly disagree) (Likert 1-2)	Neither agree nor disagree (Likert 3)	Agree (moderately agree/strongly agree) (Likert 4-5)
(i) The evolution of the research, given that sometimes there are subsequent findings that are proven uncertain or irreproducible, may reduce the credibility of the CPG recommendations	96	20 (20.8)	5 (5.2)	71 (74.0)
(ii) The complexity of the process recommended in the CPG difficults adherence	96	48 (50.0)	6 (6.3)	42 (43.8)
(iii) Scientific advances organized in the form of guidelines and recommendations are an invaluable help for clinicians	98	2 (2.0)	2 (2.0)	94 (95.9)^∗^
(iv) The objective of the guidelines is to provide an up-to-date informative framework that helps the clinician to make the most appropriate decisions individually for each patient	98	3 (3.0)	1 (1.0)	94 (95.9)^∗^
(v) The dynamic nature of scientific knowledge implies the periodic reassessment of the CPGs	98	2 (2.0)	0	96 (97.9)^∗^
(vi) An effective dissemination of the CPGs and their updates is necessary	98	2 (2.0)	0	96 (97.9)^∗^
(vii) There are different CPGs whose recommendations do not coincide	96	13 (13.6)	6 (6.3)	77 (80.2)^†^
(viii) Although a guide is well implemented it is difficult to maintain it, since after a certain time professionals tend to return to their previous routines	96	66 (68.8)	6 (6.3)	24 (25.0)
(ix) It is crucial to incorporate adherence indicators to the CPGs	96	4 (4.2)	6 (6.3)	86 (89.6)^†^
(x) The large number of CPGs on diabetes may prevent effective dissemination	96	6 (6.3)	4 (4.2)	86 (89.6)^†^

^∗^Consensus achieved in the first Delphi round; ^†^consensus achieved in the second Delphi round.

**Table 2:** Factors related to the healthcare system and the healthcare center.

Items of the questionnaire	Total number	Number of responses (%)		
		Disagree (moderately disagree/strongly disagree) (Likert 1-2)	Neither agree nor disagree (Likert 3)	Agree (moderately agree/strongly agree) (Likert 4-5)
Healthcare system-related factors				
(i) The limitations to the prescription established by the public healthcare system prevent treatment according to the CPG	96	18 (18.7)	3 (6.3)	72 (75.0)^†^
(ii) Differences in administrative limitations of local authorities between autonomous communities may have a different impact on adherence to CPGs	98	4 (4.1)	5 (5.1)	89 (90.9)^∗^
(iii) Recommendations of international CPGs generally do not coincide with the current situation of our healthcare system	96	45 (46.9)	10 (10.4)	41 (42.7)
Healthcare center-related factors				
(i) The clinician does not have enough time in the care of his/her patients to follow some recommendations	96	4 (4.1)	3 (3.1)	89 (92.7)^†^
(ii) There are no adequate material resources for the diagnosis and treatment recommended in the CPG	96	30 (31.3)	14 (14.6)	52 (54.2)
(iii) There are not adequate human resources for the diagnosis and treatment recommended in the CPG	96	10 (10.4)	3 (3.1)	83 (86.5)^†^

^∗^Consensus achieved in the first Delphi round; ^†^consensus achieved in the second Delphi round.

**Table 3:** Factors related to diabetes and the clinician.

Items of the questionnaire	Total number	Number of responses (%)		
		Disagree (moderately disagree/strongly disagree) (Likert 1-2)	Neither agree nor disagree (Likert 3)	Agree (moderately agree/strongly agree) (Likert 4-5)
Diabetes-related factors				
(i) The complexity of the pathology makes it difficult to be compliant with the CPG	96	61 (63.5)	11 (11.5)	24 (25.0)
(ii) The risk of hypoglycemia continues to be a limiting factor for the comprehensive control of patients with diabetes	96	12 (12.5)	4 (4.2)	80 (83.4)^†^
Clinician-related factors				
(i) Therapeutic inertia means that despite knowing the CPG recommendations, the clinician continues with his previous practice	96	12 (12.5)	4 (4.2)	80 (83.3)^†^
(ii) The constant updates of the CPGs make it difficult to be up to date and have a deep knowledge of them	96	28 (29.2)	8 (8.3)	60 (62.5)
(iii) Professionals must handle complex pharmacological treatment, which is perceived as a difficulty for intensification	96	24 (25.0)	12 (12.5)	60 (62.5)
(iv) The lack of connection between all the members of the interdisciplinary team that manages diabetes makes access to new agents and combined therapies difficult	96	23 (23.9)	10 (10.4)	63 (65.6)
(v) It would be necessary to have the support of dietitians and podiatrists in the management of patients with T2DM	98	1 (1.0)	3 (3.1)	94 (95.9)^∗^
(vi) The insufficient number of nursing personnel with specific training in diabetes education makes it difficult to approach patients with T2DM	98	2 (2.0)	2 (2.0)	94 (95.9)^∗^

^∗^Consensus achieved in the first Delphi round; ^†^consensus achieved in the second Delphi round.

## Data Availability

The data used to support the findings of this study are available from the corresponding author upon request.
