# Supplementary material for: Factors Associated with Adherence to Clinical Practice Guidelines for Patients with Type 2 Diabetes Mellitus: Results of a Spanish Delphi Consensus
Source: J Diabetes Res. 2021 Oct 23;2021:9970859. doi: 10.1155/2021/9970859 (PMC8557084; doi:10.1155/2021/9970859)
Supplement: Supplementary Materials [file 9970859.f1.docx]

**Study Questionnaire**

# SECTION 1: INVESTIGATOR’S DATA Sex:

- Men
- Woman

Age: years.

- 1. Years of professional practice years (excluding fellowship years)
  2. Have you participated in any training program on diabetes in the last 12 months?
- Yes
- No
  1. Do you participate or have you participated in any research project on diabetes, in addition to the present study, in the last 12 months?
- Yes
- No
  1. What are the clinical practice guidelines (CPG) and recommendations for the management of type 2 diabetes that you know? (please, mark all necessary)
- *ADA/EASD Clinical guidelines*
- Document of integral approach of type 2 diabetes of the SEEN (Spanish Society of Endocrinology and Nutrition)
- *AACE/ACE comprehensive type 2 diabetes management algorithm*
- Recommendations for the pharmacological treatment of hyperglycemia in type 2 diabetes. SED (Spanish Society of Diabetes)
- *Type 2 diabetes in adults: management NICE*
- *Clinical Practice Guidelines for the Prevention and Management of Diabetes in Canada. Professional Section of Diabetes Canada*
- Others. Specify………………………………….
- None
  1. What is the clinical practice guideline (CPG) that you use for the management of type 2 diabetes?
- *ADA/EASD Clinical guidelines*
- Document of integral approach of type 2 diabetes of the SEEN (Spanish Society of Endocrinology and Nutrition)
- *AACE/ACE comprehensive type 2 diabetes management algorithm*
- Recommendations for the pharmacological treatment of hyperglycemia in type 2 diabetes. SED (Spanish Society of Diabetes)
- *Type 2 diabetes in adults: management NICE*
- *Clinical Practice Guidelines for the Prevention and Management of Diabetes in Canada. Professional Section of Diabetes Canada*
- Others. Specify………………………………….
- None

**SECTION 2. PARTICIPANT’S HEALTHCARE CENTER**

- 1. In which province is your center located? ___________

7. Assistance level of your Healthcare center

- Level I
- Level II
- Level III
  1. How many endocrinologists work in your service?
     __________ endocrinologists
  2. Area in which your center is located:
- Rural area (< 5,000 inhabitants)
- Semirural area (5,000-15,000 inhabitants)
- Urbano (> 15,000 inhabitants)
  1. Where do you exercise your professional activity?
- In a public center
- In a private center
- In a mixed/public private center
  1. How many patients had you attended in the last week?:
- Less than 25
- From 25 to 75
- From 76 to 150
- More than 150
  1. Approximately, what percentage of patients attended in the last week had type2 diabetes mellitus?
- < 1%
- 2-10%
- 11-25%
- >25%
  1. Of the following factors, which are the ones that most influence you not to follow the clinical practice guidelines (CPG)? Please order them from highest to lowest influence, with 8 being the most influential and 1 being the least influencing.

| Reasons | Score |
| --- | --- |
| Administrative barriers related  to price |  |
| Direction orders |  |
| There are not sufficient material resources |  |
| There are not sufficient human resources |  |
| Lack of time |  |
| Yherapeutic inertia |  |
| Establish individualized patient management |  |
| Clinical practice guidelines (CPG) are too complicated |  |

**SECTION 3. FACTORS RELATED TO THE CLINICAL PRACTICE GUIDELINE (CPG)**

|  | Strongly disagree (1) | Moderately disagree (2) | Neither agree nor disagree  (3) | Moderately  agree (4) | Strongly agree (5) |
| --- | --- | --- | --- | --- | --- |
| The evolution of the research, given that sometimes there are subsequent findings that are proven uncertain or irreproducible, may reduce the credibility of the CPG recommendations |  |  |  |  |  |
| The complexity of the process recommended in the CPG difficults adherence |  |  |  |  |  |
| Scientific advances organized in the form of guidelines and Recommendations are an invaluable help for clinicians |  |  |  |  |  |
| The objective of the guidelines is to provide an up-to-date informative framework that helps the clinician to make the most appropriate decisions individually for each patient. |  |  |  |  |  |
| The dynamic nature of scientific knowledge implies the periodic reassessment of the CPGs |  |  |  |  |  |
| An effective dissemination of the CPGs and their updates is necessary |  |  |  |  |  |
| There are different CPGs whose recommendations do not coincide |  |  |  |  |  |
| Although a guide is well implemented it is difficult to maintain it, since after a certain time professionals tend to return to their previous routines |  |  |  |  |  |
| It is crucial to incorporate adherence indicators to the CPGs |  |  |  |  |  |
| The large number of CPGs on diabetes may prevent effective dissemination |  |  |  |  |  |

**SECTION 4. FACTORS RELATED TO THE HEALTHCARE SYSTEM**

|  | Strongly disagree (1) | | Moderately disagree (2) | Neither agree nor disagree  (3) | Moderately  agree (4) | Strongly agree (5) |
| --- | --- | --- | --- | --- | --- | --- |
| The limitations to the prescription established by the public healthcare system prevent treatment according to the CPG | |  |  |  |  |  |
| Differences in administrative limitations of local authorities between autonomous communities may have a different impact on adherence to CPGs | |  |  |  |  |  |
| Recommendations of International CPGs generally do not coincide with the current situation of our healthcare system | |  |  |  |  |  |

**SECTION 5. FACTORS RELATED TO THE HEALTHCARE CENTER**

|  | Strongly disagree (1) | Moderately disaagree (2) | Neither agree nor disagree (3) | Moderately agree (4) | Strongly agree  (5) |
| --- | --- | --- | --- | --- | --- |
| The clinician does not have enough time in the care of his/her patients to follow some recommendations |  |  |  |  |  |
| There are no adequate material resources for the diagnosis and treatment recommended in the CPG |  |  |  |  |  |
| There are not adequate human resources for the diagnosis and treatment recommended in the CPG |  |  |  |  |  |

**SECTION 6. FACTORS RELATED TO DIABETES**

|  | Strongly disagree (1) | Moderately disagree (2) | Neither agree nor disagree  (3) | Moderately  agree (4) | Strongly agree (5) |
| --- | --- | --- | --- | --- | --- |
| The complexity of the pathology makes it difficult to be compliant with the CPG |  |  |  |  |  |
| The risk of hypoglycemia continues to be a limiting factor for the comprehensive control of patients with diabetes |  |  |  |  |  |

**SECTION 7. FACTORS RELATED TO THE CLINICIAN**

|  | Strongly disagree (1) | Moderately disagree (2) | Neither agree nor disagree  (3) | Moderately  agree (4) | Strongly agree (5) |
| --- | --- | --- | --- | --- | --- |
| Therapeutic inertia means that despite knowing the CPG recommendations, the clinician continues with his previous practice |  |  |  |  |  |
| The constant updates of the CPGs make it difficult to be up to date and have a deep knowledge of them |  |  |  |  |  |
| Professionals must handle complex pharmacological treatment, which is perceived as a difficulty for intensification |  |  |  |  |  |
| The lack of connection between all the members of the interdisciplinary team that manages diabetes makes access to new agents and combined therapies difficult |  |  |  |  |  |
| It would be necessary to have the support of dietitians and podiatrists in the management of patients with T2DM |  |  |  |  |  |
| The insufficient number of nursing personnel with specific training in diabetes education makes it difficult to approach patients with T2DM |  |  |  |  |  |

**SECTION 8. FACTORS RELATED TO THE PATIENT**

|  | Strongly disagree (1) | Moderately disagree (2) | Neither agree nor disagree  (3) | Moderately  agree (4) | Strongly agree (5) |
| --- | --- | --- | --- | --- | --- |
| Patient characteristics do not exactly match those specified in the CPG |  |  |  |  |  |
| Patient preferences do not coincide with the CPG recommendations |  |  |  |  |  |
| The patient does not tolerate the action recommended by the CPG, as a consequence an alternative intervention must be performed |  |  |  |  |  |
| The concurrence of comorbidities in the patient makes the application of CPG difficult |  |  |  |  |  |
| In polypathological patients, it is difficult to adapt CPG |  |  |  |  |  |
| Complexity of treatment makes adherence difficult |  |  |  |  |  |
| The difficulty of the patient to follow compliance with the hygienic-dietetic and lifestyle recommendations prevents achieving the therapeutic objectives |  |  |  |  |  |
